# Sharing the initial experience of pan-cancer panel analysis in high-risk renal cell carcinoma in the Korean population

**DOI:** 10.1186/s12894-020-00687-2

**Published:** 2020-08-18

**Authors:** Jungyo Suh, Chang Wook Jeong, Seongmin Choi, Ja Hyeon Ku, Hyeon Hoe Kim, Kwangsoo Kim, Cheol Kwak

**Affiliations:** 1 Department of Urology, Seoul National University College of Medicine, Seoul, South Korea; 2 Hospital Medicine Center, Seoul National University Hospital, Seoul, South Korea; 3 Department of Medicine, Seoul National University College of Medicine, Seoul, South Korea; 4 Transdisciplinary Department of Medicine & Advanced Technology, Seoul National University Hospital, Seoul, South Korea

**Keywords:** Kidney cancer, Genes, VHL gene, PBRM1 mutation, Metastasis, Pan-cancer panel, Next-generation sequencing

## Abstract

**Background:**

This study aimed to assess the feasibility of a pan-cancer panel assay for high-risk renal cell carcinoma (RCC) in the Korean population. We also analyzed the clinical and genetic factors contributing to metastasis in clear cell RCC.

**Methods:**

Thirty-one patients with advanced RCC who underwent radical nephrectomy were analyzed. A 1.8 Mb multi-cancer panel (including 25 RCC-related genes, such as VHL, PBRM1, SETD2, and MET), comprising 181 target genes, 23 fusion genes, and 45 drug target lesions developed by Seoul National University Hospital, was used for this study.

**Results:**

We extracted DNA from 30 of the 31 (96.7%) RCC specimens. Twenty-one patients (average age 63.3 ± 11.3 years) with clear cell RCC, 5 with papillary RCC, 3 with chromophobe RCC, and one patient, each with MiT family translocation carcinoma RCC and succinate dehydrogenase deficiency RCC, were analyzed. The sequencing depth was 430.8 ± 206.6 and 97 mutations (7.3 ± 2.7 mutations per patient) were detected. The most commonly mutated genes were *VHL* (46%), *PBRM1* (30%), and *BAP1*, *NOTCH4*, and *POLQ* (23.33% each). Compared with TNM stage matched data from TCGA of clear cell RCC, *VHL* and *PBRM1* are most common in both cohorts. Univariate and multivariate analyses revealed that tumor size (Hazard ratio = 2.47, *p* = 0.04) and *PBRM1* (Hazard ratio = 28.69, *p* = 0.05) were related to metastasis in clear cell RCC.

**Conclusion:**

The pan-cancer panel comprised of RCC-related genes is a feasible and promising tool to evaluate genetic alterations in advanced RCC. However, large-scale studies and a focus on the clinical utility of this cancer panels is needed.

## Background

Renal cell carcinoma (RCC) is a malignancy that arises in the nephron tubules and has very heterogeneous histologic and clinical manifestations, accounting for approximately 90% of all cases of kidney cancer and 2.4% of all adult tumors [1]. The incidence of RCC continues to increase [2]. As of 216, there are more than 15 RCC subtypes classified by the World Health Organization [2], based on histologic and molecular criteria. Clear cell RCC (ccRCC) is the most common subtype, accounting for 75% of RCC cases, followed by papillary RCC (pRCC) and chromophobe RCC (chrRCC) [3]. The MiT family of translocation carcinomas (tRCC) and succinate dehydrogenase deficiency RCC (SDHD RCC) are rare subtypes, which are diagnosed based on molecular alterations.

Recent advances in next-generation sequencing (NGS) technology have revealed numerous genetic alterations that are important in RCC pathogenesis and prognosis [4–6]. For instance, the driver mutation of each subtype, such as von Hippel-Lindau (VHL), PBRM1, and BRCA-1 associated tumor protein 1 (BAP1) in ccRCC; MET and fumarate hydratase (FH) in pRCC; and phosphatase and tensin homolog (PTEN) or TP53 in chrRCC; as well as the mutations related to prognosis, such as BAP1, PBRM1, or SET domain-containing 2 (SETD2) in ccRCC and cyclin-dependent kinase inhibitor 2A (CDKN2A) in pRCC have been identified in numerous NGS studies [7]. In the era of precision medicine, determining the molecular alteration in each RCC by NGS analysis is essential for diagnosis and treatment planning. However, Asian data have been limited in previously conducted large-scale NGS studies [8] and the histologic subtype and clinical behavior of RCC can differ among races [9]. Thus, Korean NGS data are essential for precision medicine.

We developed a pan-cancer panel to screen for important genetic alterations in various solid tumors, including major urological cancers, such as prostate, kidney, and bladder cancers. We assessed the feasibility of the pan-cancer panel assay for high-risk RCC in the Korean population. We also analyzed the clinical and genetic factors contributing to metastasis in ccRCC to determine the clinical utility of the pan-cancer panel assay for high-risk ccRCC.

## Methods

### Patient selection

All patients were selected from the Seoul National University Prospectively Enrolled Registry for Renal Cell Carcinoma – Nephrectomy (SUPER-RCC-Nx). This is a prospective, multidisciplinary, and biobank lined cohort that was established in March 2016 [10]. This prospective cohort collects patients’ preoperative information, pathologic reports, surgical procedure details, and information on postoperative complications functional outcomes, and oncological outcomes. We selected 31 advanced RCC patients who were pathologically T3–4 or N1 or M1. The patients had undergone radical nephrectomy surgery from March 2016 to June 2016. The tumor and normal tissues that were collected in the operating room or frozen biopsy room were immediately stored in a − 195 °C liquid nitrogen tank at the SNUH Cancer Tissue Bank.

### Cancer panel

The FIRST-panel version 3 and 3.1 SNUH cancer panel was used for this analysis. The panel was developed by SNUH. It includes all exons of 183 genes, specific introns of 23 fusion genes, the telomerase reverse transcriptase promoter region, 8 microsatellite instability (MSI) markers, and 45 drug target lesions. The total length captured was approximately 1.949 Mbp. The FIRST-panel was designed to screen for the important genetic alterations in major urological malignancies, including prostate, bladder, and kidney cancers [11]. We selected RCC-related mutations by reviewing landmark studies, and finally selected 25 renal cell carcinoma-related genes, including VHL, PBRM1, SETD2, and MET mutations, in the FIRST-panel version 3.x.

### DNA extraction from fresh frozen tissue

Fresh frozen tumor tissues were homogenized and lysed with proteinase K. Total DNA was isolated from each target using the Maxwell® 16 CSC DNA Blood kit (Promega Corp., Madison, WI, USA). Extracted DNA was quantitated using a Quantus fluorometer (Promega Corp.) and TapeStation4200 (Agilent Technologies, Santa Clara, CA, USA).

### Capture library preparation and sequencing

The quality of functional genomic DNA was assessed using the 2200 TapeStation System (Agilent Technologies) before preparation of the library. The input DNA (200 ng ~ 1 μg) was sheared using an S220 Focused-ultrasonicator (Covaris, Inc., Woburn, MA, USA). Paired-end libraries were prepared with the SureSelectXT Target Enrichment System Kit (Agilent Technologies) for the Illumina paired-end sequencing library protocol using SNUH FIRST Cancer Panel v3.0 and v.3.1, according to the manufacturer’s instructions. The quality of the DNA library was evaluated using a Bioanalyzer 2100 and DNA 1000 chips (Agilent Technologies). The final libraries were sequenced on the Illumina Hiseq 2500 platform (2× 100 bp and 1000× coverage).

### Next-generation sequencing

Targeted NGS was performed using the Illumina Hiseq 2500 platform. Sequencing data were transformed as FASTQ files and quality control (QC) by FASTQC and Trimmomatic (0.33). Binary alignment/map (BAM) formation was performed after alignment based on the reference genome (GRCh 37) by BWA (0.7.12) and Picard (1.134). QC of BAM files was performed using SAMtools (v1.2) and GATK (v3.3). Single nucleotide polymorphism (SNP) discovery was performed using MuTect (1.1.7) and SAMtools (v1.2). Indel and copy number variation (CNV) discovery were performed by IndelGeontyper (0.36.3336) and CoNIFER (0.2.2), respectively. The fusion search was conducted using Delly (0.7.2). All data were converted to VCF format and annotated using ANNOVAR.

### Variant prevalence comparison of SNUH pan-cancer data and TCGA prostate cancer data

Three RCC databases were downloaded from The Cancer Genome Atlas (TCGA; TCGA-KIRC, TCGA-KIRP, TCGA-KICH) variant MAF files, belonging to the NCI GDC data portal. We extracted the variants of interest from the MAF files: *VHL, PBRM1, BAP1, SETD2, KMT2D, MET, TP53, FH, BRCA2, TSC1, TSC2, KMT2D, NOTCH3, NOTCH*4, DNA polymerase theta (*POLQ*), Franconi anemia complementation group A (*FANCA*), and ataxia telangiectasia and Rad3-related (ATR). Four MAF files were provided for their corresponding variant callers: MuSE, Mutect2, VarScan2, and SomaticSniper. We selected variants that were present in at least two of these files. According to the “Variant_Classification” column of the MAF files, the data were categorized as missense mutation, truncating mutation (frameshift indels, splice site variants, and nonsense mutations), and in-frame indels. After matching pathologic TNM staging (T3 over or N1 or M1) of TCGA data with SNUH data, the prevalence of the mutations was compared with our SNUH pan-cancer data.

### Integrative statistical analyses of clinical and genomic data

The clinical and demographic variables of three of the major subtypes of RCC were compared. As the tRCC and SDHD RCC groups contained one patient each, only described characteristics were compared, without statistical analysis. The continuous variables are summarized as the median value, and categorical variables are reported as actual numbers and percentages. A one-way ANOVA test was performed for three of the major groups (ccRCC, pRCC, and chrRCC).

We performed univariate and multivariate analyses of clinical and genomic data from 20 ccRCC patients. The factors associated with the presence of metastasis (both synchronous and metachronous) were compared using Student’s t-test, Pearson Chi-square test, or Fischer’s exact test. Univariate and multivariate logistic regression analyses were then performed for the presence of metastasis. All statistical analyses were conducted using SPSS Statistics 22 (IBM, Armonk, NY, USA). A *p*-value < 0.05 was considered significant.

## Results

### Patient characteristics

The demographic and clinical characteristics of the enrolled patients are presented in Table 1. The 31 patients were categorized into the following subclasses of RCC: ccRCC (*n* = 21), pRCC (*n* = 5), chrRCC (*n* = 3), tRCC (*n* = 1), and SDHD RCC (n = 1). Our data only contained type 2 subtypes of pRCC. The mean follow-up period was 19.0 months. Twelve patients presented with metastatic lesions within the follow-up period, including 6 synchronous and 6 metachronous metastases. There were no deaths in the follow-up period.

**Table 1:** Baseline characteristics of five types of RCC evaluated by this study

Characteristics	Clear cell *N* = 20	Papillary *N* = 5	Chromophobe *N* = 3	*P*-value	tRCC *N* = 1	SDHD N = 1
Age (range) - yr	63.6 ± 11.3	67.6 ± 10.2	63.0 ± 8.66	0.752	60	39
Sex – male (%)	13 (65)	4 (80)	2 (66.7)	0.830	1 (100)	0 (0)
BMI (kg/m^2^)	24.9 ± 5.1	25.2 ± 3.4	24.6 ± 2.4	0.985	22.60	24.20
DM	6 (30)	2 (40)	0 (0)	0.495	1 (100)	0 (0)
HTN	12 (60)	3 (60)	2 (66.7)	0.978	1 (100)	0 (0)
Mass size	7.04 ± 3.1	7.08 ± 4.3	7.67 ± 4.0	0.434	10.9	2.5
Pathologic T stage – N (%)				0.927		
< T2	2 (10)	1 (20)	0 (0)		0 (0)	0 (0)
T3	17 (85)	4 (80)	3 (100)		1 (100)	1 (100)
T4	1 (5)	0 (0)	0 (0)		0 (0)	0 (0)
Tumor thrombus level– N (%)				0.354		
Level 1	3 (15)	0 (0)	1 (33.3)		0 (0)	0 (0)
Level 2	1 (5)	1 (25)	0 (0)		1 (100)	0 (0)
Fuhrman grade				0.830		
2	0 (0)	0 (0)	0 (0)		0 (0)	1 (100)
3	19 (95)	5 (100)	3 (100)		1 (100)	0 (0)
4	1 (5)	0 (0)	0 (0)		0 (0)	0 (0)
Pathologic N stage – N (%)				–		
N1	0 (0)	0 (0)	0 (0)		1 (100)	0 (0)
Pathologic M stage – N (%)				0.642		
M1	4 (20)	1 (20)	0 (0)		1 (16.7)	0 (0)

### Sequencing QC

DNA was successfully extracted from the 30 fresh frozen tissue samples (96.8%) for library preparation. DNA could not be extracted from a sample with 90% necrotic lesion (ccRCC) in the library preparation step. The total read number ranged from 22,704,326 to 35,508,848, and the average size of FASTQ data was 2084.7 ± 265.3 Mb in the cancer panel analysis of the 30 patients. The average sequencing depth was 430.8 ± 206.6, ranging from 152 to 971. Average values of coverage above 50 and 100 were 98.6 ± 3.5% and 96.4 ± 5.8%, respectively.

### Pan-cancer panel report

**Fig. 1:**
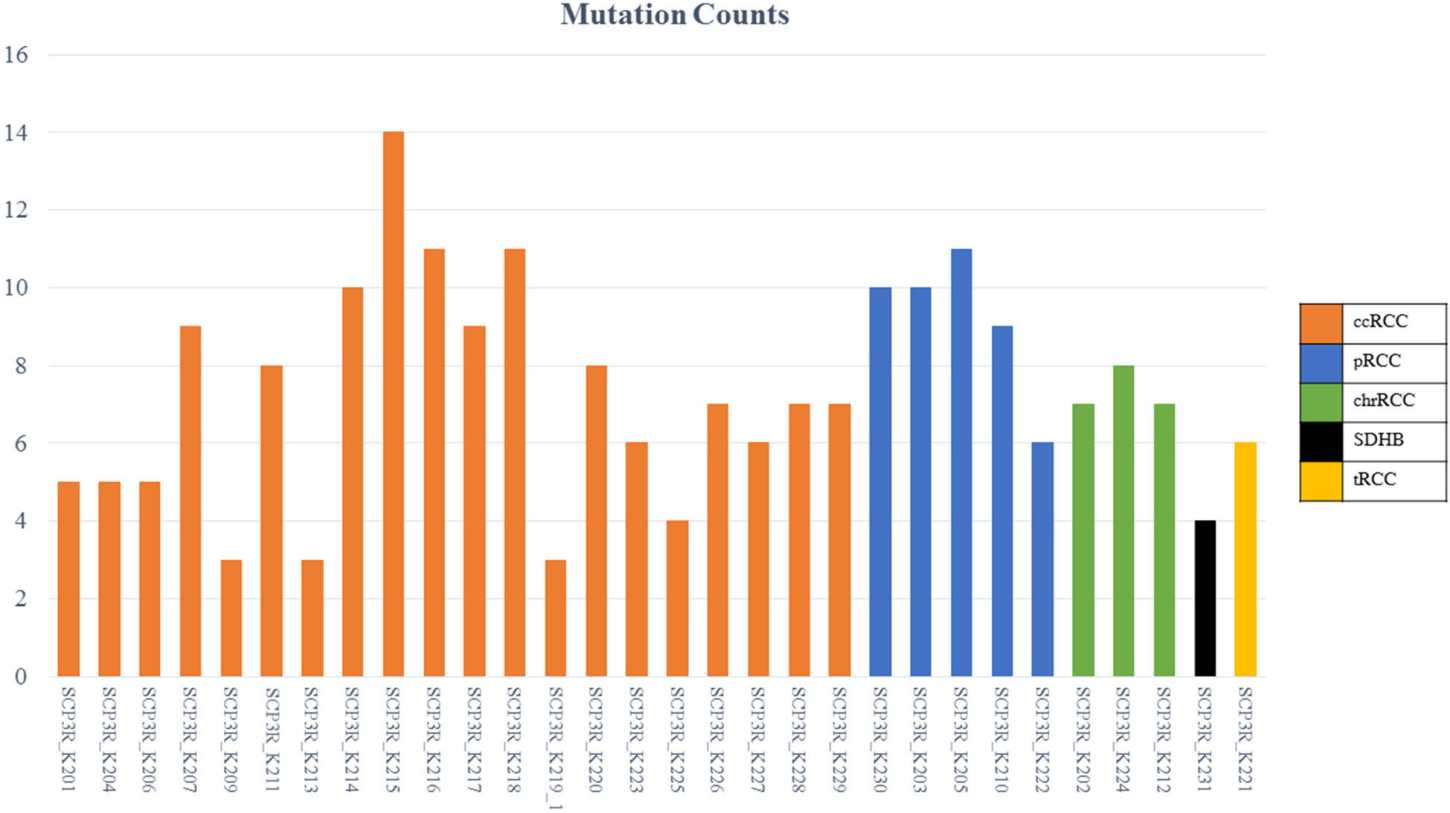
Specific genetic alteration counts of each RCC patient. Minimum 3 to maximum 14 mutations per each patient was founded by pan-cancer panel analysis. Each bar shows SNP or INDEL mutations. There were no structural variations

**Fig. 2:**
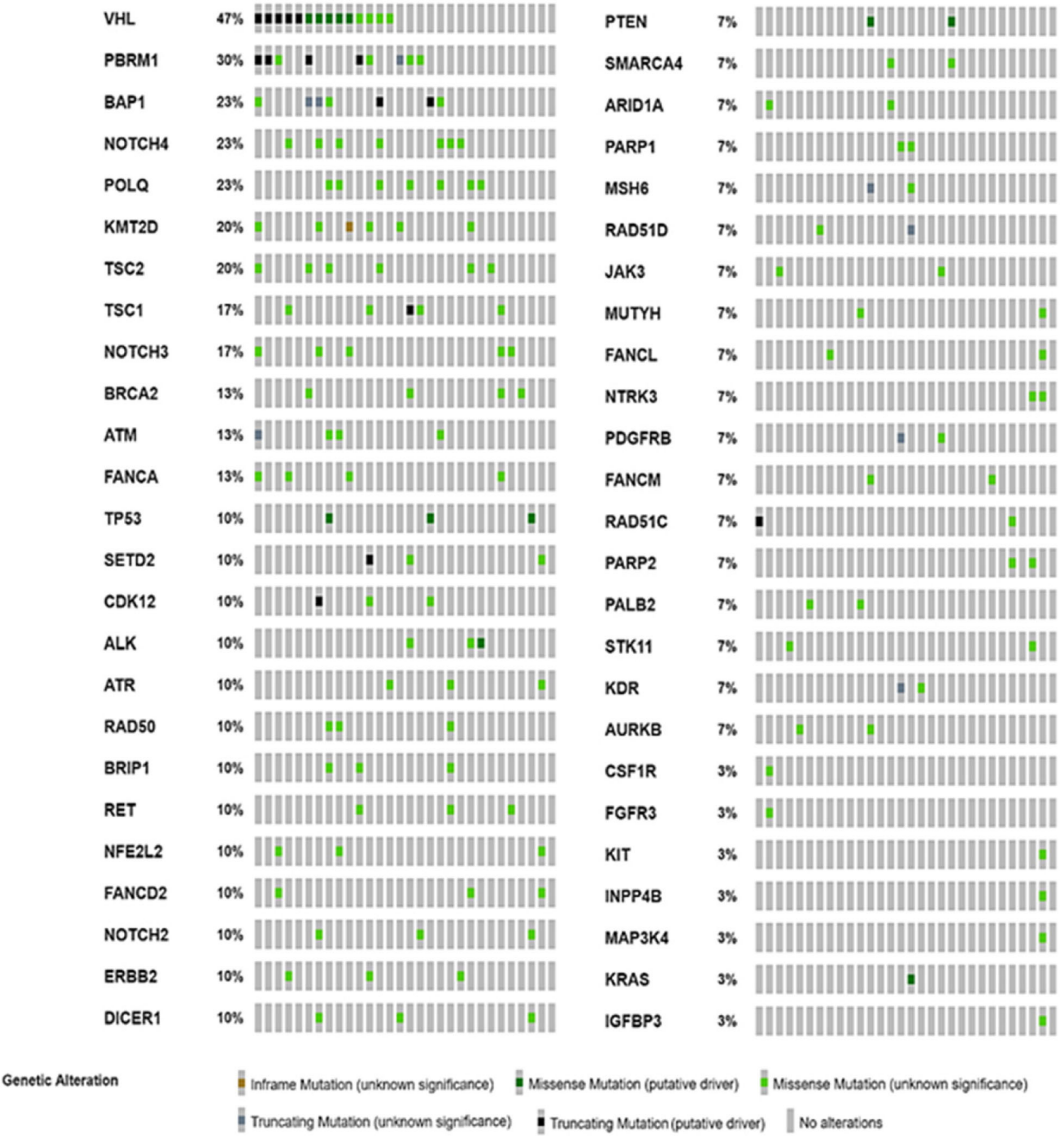
Integrative analysis of pan-cancer panel analysis of 30 RCC patients. Each grey column represented a specific 30 patient data in order. This oncoprint obtained by The cBioPortal for Cancer Genomics (http://cbioportal.org) graphic visualization tool

A total of 97 mutations were detected in the pan-cancer panel analysis. *VHL* was the most commonly mutated gene (46.67%). This observation was evident only in ccRCC. Genetic alterations in *PBRM1* (30%), *NOTCH4*, *POLQ*, and *BAP1* (23.3% each) were also frequently detected. An average of 7 SNPs was detected per patient. However, no fusion or CNVs were noted (Fig. 1). Integrative analysis of the pan-cancer panel data are presented in Fig. 2. We detected *SDHB* mutation in the specimen obtained from the SDHD RCC patient. The *TFE3* mutation was detected in the specimen of a tRCC patient. None of the mutations detected was exclusive to a particular subtype of RCC. No mutation common to all mutational profiles was detected.

### Comparison with TCGA database

**Fig. 3:**
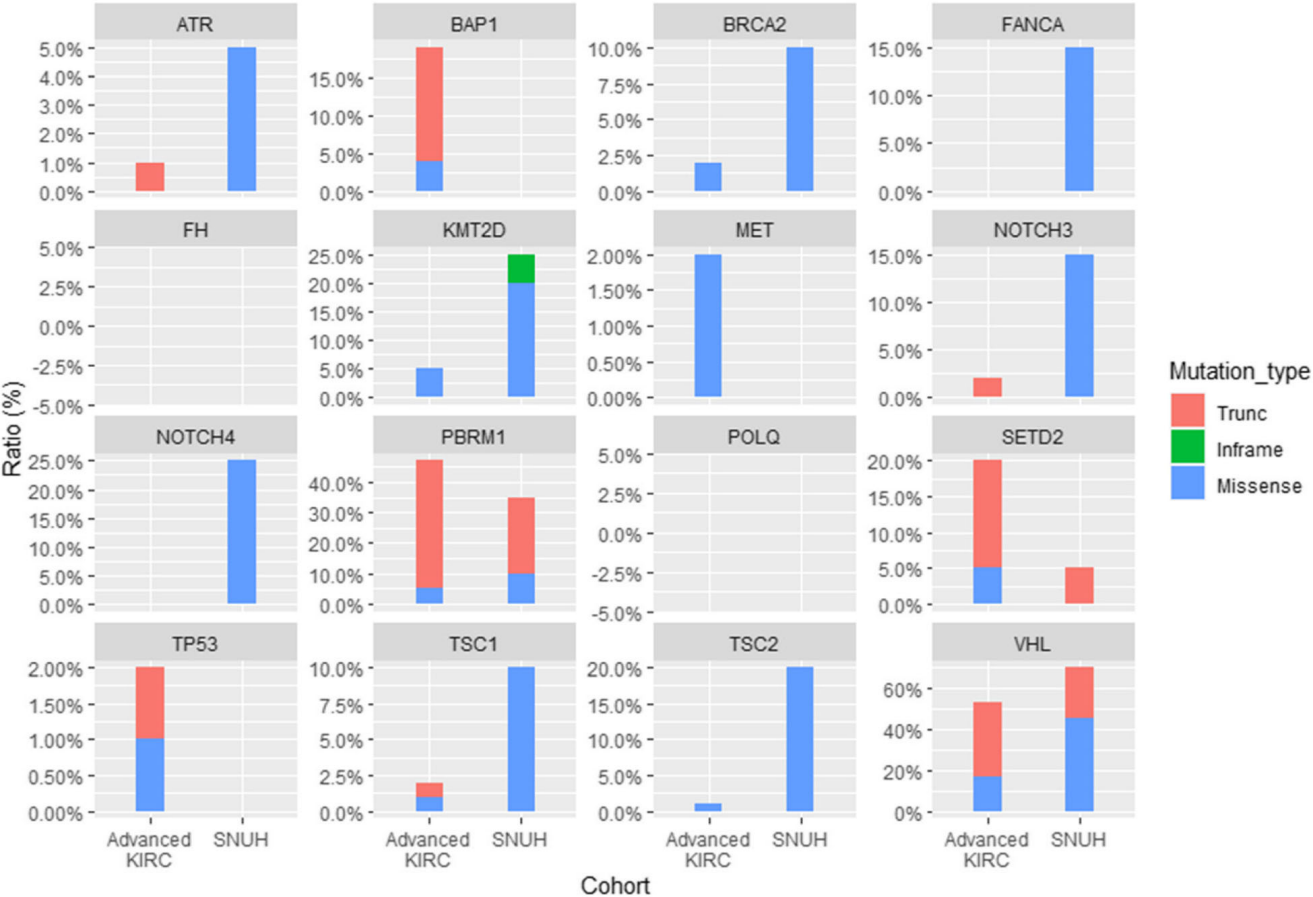
The comparison of FIRST panel result to TNM stage matched TCGA cohort for clear cell RCC

We compared the SNUH database with 110 TNM stage matched advanced RCC data from TCGA (TCGA-advance KIRC). The 17 mutations of interest (*VHL, PBRM1, BAP1, SETD2, KMT2D, MET, TP53, FH, BRCA2, TSC1, TSC2, KMT2D, NOTCH3, NOTCH4, POLQ*, *FANCA*, and *ATR*) were compared between the two groups. *VHL* (70.0% vs. 59.0%) and *PBRM1* (35.0% vs. 52.0%) were prevalent in both groups. *KMT2D, TSC1, TSC2, NOTCH3, NOTCH4, BRCA2, FANCA,* and *ATR* were more frequently mutated in the FIRST-panel analysis of RCC, compared to the TCGA-advance KIRC database. However, mutations in *PBRM1*, *SETD2*, and *BAP1* were more common in the TCGA-advance KIRC database. No mutations were detected in *POLQ*, *BAP1*, *MET*, *TP53*, and *FH* genes in the FIRST-panel analysis for ccRCC (Fig. 3)

.

The FIRST-panel results revealed that *PBRM1*, *POLQ*, *TSC1*, and *SETD2* genes were commonly mutated in papillary RCC. The MET mutation was frequently detected in the TCGA-KIRP database. However, no mutation or CNVs were observed in the FIRST-panel analysis of RCC patients. The type 1 and 2 pRCC displayed different molecular signatures, and MET mutations were more frequently found in the type 1 subtype. All five patients were the type 2 subtype, featuring commonly altered *CDKN2A, SETD2, BAP1*, and *PBRM1* mutations, consistent with our data. In the chromophobe subtype, the TP53 mutation was common in TCGA-KICH database. However, FIRST-panel analysis did not reveal any mutations in the TP53 gene in this subtype. A detailed comparison of the FIRST-panel and TCGA database was performed for papillary RCC and chromophobe RCC (Supplementary Table 1). Owing to the limited number of mutations included in the FIRST-panel, we could not examine some of the important mutations of RCC (*TTN*, *MUC4*, and *MUC16*), which are relatively common in the TCGA database.

### Univariate and multivariate analyses of metastatic events

Clinical and pathologic information concerning the presence of a metastatic event was compared (Table 2). Mass size (*p* = 0.008) was significantly different between metastatic and localized RCC. In the mutation profile data, only *PBRM1* showed a possible relationship with metastasis. (*p* = 0.062). Univariate and multivariate logistic regression analyses data for metastasis are presented in Table 3. Among the clinicopathologic information and mutation data, only tumor size (*p* = 0.042) and *PBRM1* (*p* = 0.046) mutations were statistically correlated with metastasis.

**Table 2:** Compare of clinicopathologic features and mutational profile by the presence of metastasis in clear cell RCC

	Localized *N* = 12	Metastatic *N* = 8	*p*-value
**Age**	61.4 ± 12.7	66.9 ± 8.6	0.303
**Sex – male (%)**	8 (66.7%)	5 (62.5%)	1.000^‡^
**BMI (kg/m**^**2**^**)**	25.7 ± 4.3	23.8 ± 6.3	0.439
**DM**	8 (66.7%)	6 (75.0%)	1.000^‡^
**HTN**	5 (41.7%)	3 (37.5%)	1.000^‡^
**Mass size (cm)**	5.8 ± 2.0	8.8 ± 2.4	**0.008**
**Pathologic T stage – N (%)**			0.071^※^
**< T2**	0 (0%)	2 (25.0%)	
**T3**	12 (100%)	5 (62.5%)	
**T4**	0 (0%)	1 (12.55)	
**Tumor thrombus level– N (%)**			0.225^※^
**Level 0**	10 (83.3%)	5 (62.5%)	
**Level 1**	1 (8.3%)	3 (37.5%)	
**Level 2**	1 (8.3%)	0 (0%)	
**Fuhrman grade**			0.400‡
**3**	12 (100%)	4 (87.5%)	
**4**	0 (0%)	1 (12.5%)	
**Lymphovascular invasion**	2 (16.7%)	4 (50.0%)	0.161‡
**Mutation Counts**	7.0 ± 3.2	7.1 ± 2.9	0.930
**VHL**	9 (75.0%)	5 (62.5%)	0.642‡
**PBRM1**	2 (16.7%)	5 (62.5%)	0.062‡
**NOTCH4**	4 (33.3%)	1 (12.5%)	0.603‡
**KMT2D**	1 (8.3%)	1 (12.5%)	1.000‡
**NOTCH3**	2 (16.7%)	1 (12.5%)	1.000‡
**TSC1**	3 (25.0%)	1 (12.5%)	0.619
**BAP1**	5 (41.7%)	2 (25.0%)	0.642‡
**BRCA2**	2 (16.7%)	0 (0%)	0.495‡
**SETD2**	0 (0%)	1 (12.5%)	0.400‡

^※^for Pearson Chi-square, ‡ for Fisher’s Exact Test

**Table 3:** Univariate and multivariable analysis of clinco-pathologic feature and mutational profile for metastasis in clear cell RCC

	Univariate	*p*-value	multivariable	*p*-value
	OR (95% CIs)		OR (95% CIs)	
Age	1.048 (0.961–1.142)	0.288		
Sex	1.200 (0.185–7.770)	0.848		
Mass size (continuous)	1.952 (1.052–3.622)	**0.034**	2.47 (1.03–5.92)	**0.042**
Pathologic T-stage		1.000		
Thrombus level		0.374		
Fuhrman Grade		1.000		
Mutation count	1.015 (0.748–1.377)	0.925		
VHL mutation	0.556 (0.080–3.858)	0.552		
PBRM1 mutation	8.333 (1.034–67.142)	**0.046**	28.39 (1.06–758.79)	**0.046**
BAP1 mutation	0.467 (0.065–3.344)	0.448		

## Discussion

In this study, we successfully identified 97 genomic alterations in various subtypes of RCC. DNA was extracted from 30 of the 31 patients’ samples. All 30 DNA samples were successfully analyzed using the pan-cancer panel. DNA could not be extracted from the fresh frozen tissue of one patient, which consisted mostly of necrotized tissue. Using univariate and multivariate analyses, we assessed the risk factors contributing to metastasis in ccRCC patients.

Most of the mutational signatures of ccRCC detected by this pan-cancer panel analysis were similar to those in the TCGA database and previous genetic studies. For example, in this study, *VHL* mutation was the most common mutation (70.0%) in ccRCC samples, followed by the PBRM1 mutation (35.0%). Genetic or epigenetic alterations in chromatin remodeling genes, which include *VHL*, *BAP1*, *PBRM1*, and *SETD2*, are the most prevalent events in the development of ccRCC [12, 13]. Similar to our findings, alterations in the *VHL* gene are reported to be the most frequent (60–70%), while those in *PBRM1*, *BAP1*, and *SETD2* were reported as 40, 10, and 10%, respectively, in previous genetic studies [4, 13, 14]. We also found *TP53* and *PTEN* mutations in chrRCC patients. These are important driver mutations of chrRCC [6, 15]. However because all the patients were papillary type 2, we did not detect MET mutations in pRCC patients in the pan-cancer analysis, which are more important driver mutation of type 1 pRCC than type 2 pRCC [15, 16].

Important driver mutations of uncommon RCC subtypes (tRCC and SDHD RCC) were also detected by the pan-cancer panel analysis. MiT family translocation carcinomas (tRCC) are characterized by translocations involving breakpoint lesions at Xp11.2 and are frequently fused with the *TFE3* gene [17, 18]. SDHD RCC is a newly classified subtype that was first announced in the 2016 WHO classification [2]. This subtype is characterized by genetic alterations in the Krebs cycle enzymes (SDHB/C/D) that result in the Warburg effect in ccRCC with the accumulation of hypoxia-inducible factor [19]. SDHD RCC can be diagnosed by distinguishing histologic features, such as vacuolated eosinophil or clear cells. However, detecting SDHB alteration by immunohistochemistry is more effective [2]. We successfully detected SDHB and TFE3 mutations in tissues specimens from SDHD RCC and tRCC patients using the pan-cancer panel analysis.

Although the mutational profile revealed by our pan-cancer panel analysis was mostly similar to the TCGA database, some discrepancies were evident. In this study, *NOTCH* family genes (*NOTCH3*/*4*), *TSC1*, *TSC2*, and *KMT2D* showed a relatively higher incidence of mutations than TNM matched data from TCGA-KIRC [4]. *BRCA* and *FANCA* is well known mutation of DNA repair pathway and is more common in FIRST panel analysis than TCGA-advanced KIRC. Owing to the small number of Asian patients included in the TGCA database [4–6], these differences may provide clues regarding the prevailing racial differences. However, owing to small number of study population, we cannot made concrete conclusion. More large-scale data are needed before any definitive conclusions can be made.

We conducted univariate and multivariate analyses using genetic alterations and clinicopathologic features in ccRCC. The multivariable analysis showed that the PBRM1 mutation and primary tumor size were significantly associated with metastasis in ccRCC. Primary tumor size is a well-established prognostic factor of metastasis [20, 21]. Eric et al. [21] retrospectively analyzed data from 2651 RCC patients, including 182 cases of synchronous metastasis, and found that the primary tumor size was significantly associated with metastasis. In general, PBRM1 mutations mutually exclusive from BAP1 mutations, [3] and both genes harbor a higher number of alterations in cases with metastatic lesions. Eckel-Passow et al. [22] analyzed paired tissue of primary and metastatic ccRCC and found that both BAP1 and PBRM1 were highly altered in metastatic lesions (98 and 90%, respectively), compared to the primary lesions (20 and 57%, respectively). The PBRM1 mutation alone is considered more favorable than the BAP1 mutation, but alterations in both lesions correlate with worst survival [23]. We could not analyze the expression pattern of BAP1 with PBRM1 or study their prognostic impact on survival, as no BAP1 mutations were detected. This observation might be attributed to the small sample size of our study.

With the advancement of NGS technology, there have been several attempts to use genetic analysis in clinical practice. Memorial Sloan Kettering-integrated mutation profiling of actionable cancer targets (MSK-IMPACT) [24] is a well-known pan-cancer NGS panel that targets more than 341 cancer associated genes. The MSK-IMPACT panel was successfully applied for a multi-institutional, diverse primary tumor prospective cohort including more than 12,670 tumor samples in 2017 [25]. These were promising results. However, the clinical application of NGS cancer panels in routine practice is not a reality yet, due to several issues of low matching yield in clinical trials [26, 27], high cost [28], and racial differences [26, 28]. The present study represents only a first step towards oncology precision medicine for clinical practice in advanced RCC. More data from global studies are required for further progress.

This study has several limitations. RCC is a highly heterogeneous disease, and the statistical power of our study (owing to the small sample size) may not be enough to concretely support our findings. In addition, because we used targeted sequencing to detect alterations in RCC, we did not determine the whole mutational profile of the RCC patients who were screened. Despite these limitations, we successfully conducted a pan-cancer panel analysis with good sequencing depth (> 400). The data shed light on the feasibility of using the pan-cancer panel for the diagnosis of RCC, and the possibility of deriving meaningful prognostic information from the mutational profiles.

## Conclusion

The pan-cancer panel comprised of RCC-related genes is a feasible and promising tool to evaluate genetic alterations in advanced RCC. However, the limited information on genetic analysis necessitates large-scale studies and a focus on the clinical utility of cancer panels to further explore the routine use of the panel.

## Supplementary information

**Additional file 1: Supplementary Table 1.** Comparison of SNUH pan-cancer panel result with TCGA database of papillary RCC and chromophobe RCC.

## Data Availability

The datasets generated during and/or analysed during the current study are not publicly available due to included patient information but are available from the corresponding author on reasonable request.

## Abbreviations

RCC: Renal cell carcinoma
ccRCC: Clear cell renal cell carcinoma
pRCC: Papillary renal cell carcinoma
chrRCC: Chromophobe renal cell carcinoma
tRCC: MiT family translocation renal cell carcinoma
SDHD: Succinate dehydrogenase deficiency
NGS: Next-generation sequencing
SNUH: Seoul National University Hospital
IRB: Institutional review board
SUPER-RCC-Nx: Seoul National University Prospectively Enrolled Registry for Renal cell carcinoma – Nephrectomy
TCGA: The Cancer Genome Atlas
