# Supplementary material for: Sharing the initial experience of pan-cancer panel analysis in high-risk renal cell carcinoma in the Korean population
Source: BMC Urol. 2020 Aug 18;20:125. doi: 10.1186/s12894-020-00687-2 (PMC7433120; doi:10.1186/s12894-020-00687-2)
Supplement: Supplementary file 1 — Additional file 1: Supplementary Table 1. Comparison of SNUH pan-cancer panel result with TCGA database of papillary RCC and chromophobe RCC. [file 12894_2020_687_MOESM1_ESM.docx]

**Supplementary Table 1.** Comparison of SNUH pan-cancer panel result with TCGA database of papillary RCC and chromophobe RCC.

| SNUH pan-caner panel | | | | | | | | pRCC  n=5 | TCGA database | | | | | | | |
| --- | --- | --- | --- | --- | --- | --- | --- | --- | --- | --- | --- | --- | --- | --- | --- | --- |
| Turncating | Turncating (%) | Inframe | Inframe | Missense | Missense (%) | **Sum** | **Sum (%)** | **Gene** | **Sum** | **Sum** | Truncating | Truncating (%) | Inframe | Inframe (%) | Missense | Missense (%) |
| 0 | 0.0% | **0** | 0.0% | **0** | 0.0% | **0** | **0.0%** | **VHL** | **4** | **1.4%** | 2 | 0.7% | 0 | 0.0% | 2 | 0.7% |
| 0 | 0.0% | **0** | 0.0% | **2** | 40.0% | **2** | **40.0%** | **PBRM1** | **13** | **4.7%** | 10 | 3.6% | 0 | 0.0% | 3 | 1.1% |
| 0 | 0.0% | **0** | 0.0% | **0** | 0.0% | **0** | **0.0%** | **NOTCH4** | **1** | **0.4%** | 1 | 0.4% | 0 | 0.0% | 0 | 0.0% |
| 0 | 0.0% | **0** | 0.0% | **2** | 40.0% | **2** | **40.0%** | **POLQ** | **4** | **1.4%** | 0 | 0.0% | 0 | 0.0% | 4 | 1.4% |
| 0 | 0.0% | **0** | 0.0% | **0** | 0.0% | **0** | **0.0%** | **BAP1** | **9** | **3.2%** | 2 | 0.7% | 0 | 0.0% | 7 | 2.5% |
| 0 | 0.0% | **0** | 0.0% | **1** | 20.0% | **1** | **20.0%** | **KMT2D** | **13** | **4.6%** | 4 | 1.4% | 0 | 0.0% | 9 | 3.2% |
| 0 | 0.0% | **0** | 0.0% | **1** | 20.0% | **1** | **20.0%** | **TSC2** | **6** | **2.1%** | 2 | 0.7% | 0 | 0.0% | 4 | 1.4% |
| 1 | 20.0% | **0** | 0.0% | **1** | 20.0% | **2** | **40.0%** | **TSC1** | **4** | **1.4%** | 2 | 0.7% | 0 | 0.0% | 2 | 0.7% |
| 0 | 0.0% | **0** | 0.0% | **1** | 20.0% | **1** | **20.0%** | **NOTCH3** | **3** | **1.1%** | 0 | 0.0% | 0 | 0.0% | 3 | 1.1% |
| 0 | 0.0% | **0** | 0.0% | **1** | 20.0% | **1** | **20.0%** | **BRCA2** | **7** | **2.5%** | 0 | 0.0% | 0 | 0.0% | 7 | 2.5% |
| 0 | 0.0% | **0** | 0.0% | **0** | 0.0% | **0** | **0.0%** | **FANCA** | **3** | **1.1%** | 0 | 0.0% | 1 | 0.4% | 2 | 0.7% |
| 0 | 0.0% | **0** | 0.0% | **1** | 20.0% | **1** | **20.0%** | **ATR** | **2** | **0.7%** | 0 | 0.0% | 0 | 0.0% | 2 | 0.7% |
| 0 | 0.0% | **0** | 0.0% | **2** | 40.0% | **2** | **40.0%** | **SETD2** | **17** | **6.0%** | 13 | 4.6% | 0 | 0.0% | 4 | 1.4% |
| 0 | 0.0% | **0** | 0.0% | **0** | 0.0% | **0** | **0.0%** | **MET** | **21** | **7.5%** | 0 | 0.0% | 0 | 0.0% | 21 | 7.5% |
| 0 | 0.0% | **0** | 0.0% | **0** | 0.0% | **0** | **0.0%** | **TP53** | **5** | **1.8%** | 1 | 0.4% | 0 | 0.0% | 4 | 1.4% |
| 0 | 0.0% | **0** | 0.0% | **0** | 0.0% | **0** | **0.0%** | **FH** | **2** | **0.7%** | 2 | 0.7% | 0 | 0.0% | 0 | 0.0% |
| SNUH pan-caner panel | | | | | | | | chrRCC  n=3 | TCGA database | | | | | | | |
| Turncating | Turncating (%) | Inframe | Inframe | Missense | Missense (%) | **Sum** | **Sum (%)** | **Gene** | **Sum** | **Sum** | Truncating | Truncating (%) | Inframe | Inframe (%) | Missense | Missense (%) |
| 0 | 0.0% | **0** | 0.0% | **0** | 0.0% | 0 | 0.0% | **VHL** | **0** | **0.0%** | 0 | 0.0% | 0 | 0.0% | 0 | 0.0% |
| 0 | 0.0% | **0** | 0.0% | **0** | 0.0% | 0 | 0.0% | **PBRM1** | **1** | **1.5%** | 0 | 0.0% | 0 | 0.0% | 1 | 1.5% |
| 0 | 0.0% | **0** | 0.0% | **1** | 33.3% | 1 | 33.3% | **NOTCH4** | **0** | **0.0%** | 0 | 0.0% | 0 | 0.0% | 0 | 0.0% |
| 0 | 0.0% | **0** | 0.0% | **0** | 0.0% | 0 | 0.0% | **POLQ** | **0** | **0.0%** | 0 | 0.0% | 0 | 0.0% | 0 | 0.0% |
| 0 | 0.0% | **0** | 0.0% | **0** | 0.0% | 0 | 0.0% | **BAP1** | **0** | **0.0%** | 0 | 0.0% | 0 | 0.0% | 0 | 0.0% |
| 0 | 0.0% | **0** | 0.0% | **0** | 0.0% | 0 | 0.0% | **KMT2D** | **2** | **3.0%** | 0 | 0.0% | 0 | 0.0% | 2 | 3.0% |
| 0 | 0.0% | **0** | 0.0% | **0** | 0.0% | 0 | 0.0% | **TSC2** | **2** | **3.0%** | 0 | 0.0% | 0 | 0.0% | 2 | 3.0% |
| 0 | 0.0% | **0** | 0.0% | **1** | 33.3% | 1 | 33.3% | **TSC1** | **2** | **3.0%** | 2 | 3.0% | 0 | 0.0% | 0 | 0.0% |
| 0 | 0.0% | **0** | 0.0% | **1** | 33.3% | 1 | 33.3% | **NOTCH3** | **1** | **1.5%** | 0 | 0.0% | 0 | 0.0% | 1 | 1.5% |
| 0 | 0.0% | **0** | 0.0% | **1** | 33.3% | 1 | 33.3% | **BRCA2** | **0** | **0.0%** | 0 | 0.0% | 0 | 0.0% | 0 | 0.0% |
| 0 | 0.0% | **0** | 0.0% | **1** | 33.3% | 1 | 33.3% | **FANCA** | **0** | **0.0%** | 0 | 0.0% | 0 | 0.0% | 0 | 0.0% |
| 0 | 0.0% | **0** | 0.0% | **1** | 33.3% | 1 | 33.3% | **ATR** | **1** | **1.5%** | 1 | 1.5% | 0 | 0.0% | 0 | 0.0% |
| 0 | 0.0% | **0** | 0.0% | **0** | 0.0% | 0 | 0.0% | **SETD2** | **1** | **1.5%** | 1 | 1.5% | 0 | 0.0% | 0 | 0.0% |
| 0 | 0.0% | **0** | 0.0% | **0** | 0.0% | 0 | 0.0% | **MET** | **0** | **0.0%** | 0 | 0.0% | 0 | 0.0% | 0 | 0.0% |
| 0 | 0.0% | **0** | 0.0% | **1** | 33.3% | **1** | 33.3% | **TP53** | **21** | **31.8%** | 9 | 13.6% | 0 | 0.0% | 12 | 18.2% |
| 0 | 0.0% | **0** | 0.0% | **0** | 0.0% | 0 | 0.0% | **FH** | **0** | **0.0%** | 0 | 0.0% | 0 | 0.0% | 0 | 0.0% |
